# LncRNA-MUF: A Novel Oncogenic Star with Potential as a Biological Marker and Therapeutic Target for Gastrointestinal Malignancies

**DOI:** 10.7150/jca.91984

**Published:** 2024-01-21

**Authors:** Yang Wang, Jialing Wang, Yihan Zhang, Hongliang Luo, Huazhao Yuan

**Affiliations:** 1 Department of Gastroenterology, The Second Affiliated Hospital, Jiangxi Medical College, Nanchang University, Nanchang 330008, Jiangxi, China.; 2 Department of Gastrointestinal Surgery, The Second Affiliated Hospital, Jiangxi Medical College, Nanchang University, Nanchang 330008, Jiangxi, China.; 3 Second School of Clinical Medicine, Jiangxi Medical College, Nanchang University, Nanchang 330008, Jiangxi, China.; 4 Department of General Surgery, Jiujiang Hospital of Traditional Chinese Medicine, Jiujiang 332007, Jiangxi, China.

## Abstract

Gastrointestinal (GI) cancers pose a significant global health challenge, characterized by a high incidence and poor prognosis. The delayed detection and occurrence of metastasis contribute to the overall low survival rates associated with these cancers. Therefore, there is an urgent need to identify novel molecular targets for effective GI cancer treatment. Recent research has shed light on the potential of long non-coding RNAs (lncRNAs) as promising targets in cancer therapy, given their strong association with carcinogenesis and profound impact on tumor development. Among these lncRNAs, lncRNA-MUF, also known as LINC00941, has emerged as a key player in oncogenic regulation, specifically implicated in the progression of various GI cancers, including esophageal, gastric, colorectal, hepatic, and pancreatic cancer. This review aims to provide an updated and focused analysis of the regulatory roles of LINC00941 in the initiation and progression of GI cancer. Our objective is to unravel the underlying molecular mechanisms through which LINC00941 influences GI cancer phenotypes both *in vivo* and *in vitro*, with a special emphasis on the key molecules and signaling pathways involved. Additionally, LINC00941 has demonstrated clinical significance in terms of clinical pathology, prognosis, and diagnosis in GI tumors, further reinforcing its potential as a novel therapeutic target.

## Introduction

Gastrointestinal (GI) cancers encompass a diverse range of malignancies affecting the digestive system, including the esophagus, stomach, liver, pancreas, and colorectum 1. These cancers present substantial challenges, with a significant global burden of morbidity and mortality 2, 3. In 2020 4, colorectal cancer ranked third worldwide, comprising 10% of total tumor cases (10.1 million new cases), followed by stomach cancer (5.6%), liver cancer (4.7%), and oesophageal cancer (3.1%). Similarly, in terms of mortality, colorectal cancer ranked second globally, accounting for 9.4% of total deaths (5.5 million deaths), followed by liver cancer (8.3%), stomach cancer (7.7%), oesophageal cancer (5.5%), and pancreatic cancer (4.7%). Despite the advancement of treatment modalities, such as radiation therapy, and immunotherapy, have made 5-9, the prognosis for patients with GI cancers remains unsatisfactory, characterized by a high risk of disease recurrence and metastasis. Consequently, there is an urgent need to gain a comprehensive understanding of the intricate molecular mechanisms governing the development and progression of GI cancers to facilitate the development of efficacious therapeutic strategies.

In recent years, non-coding RNAs (ncRNAs), particularly long non-coding RNAs (lncRNAs), have emerged as important players in cancer biology 10-14. LncRNAs have been found to be involved in a variety of biological processes, can act as oncogenes or tumor suppressors, and are associated with various aspects of cancer, including tumorigenesis, metastasis, and treatment resistance 15-19. The aberrant expression patterns of lncRNAs in GI cancers have been linked to clinical outcomes, making them promising tumor markers and potential therapeutic targets 11, 20-26.

In the field of GI cancer research, considerable attention has been focused on the lncRNA-MUF, or MSC-upregulated factor. This particular lncRNA is also identified as Long Intergenic Non-Protein Coding RNA 941 (LINC00941). The LINC00941 gene is located at the 12p11.21 position within the human genome and is distinct in having just a single transcript. This singular transcript feature of the gene highlights its unique molecular characteristics in GI cancer studies. Recently, a large number of studies have shown that it is involved in the progression of various tumors 27-34, particularly in GI malignancies 35-47. Dysregulated expression of LINC00941 has been observed in GI tumors 35, 43, 44, 46. Functionally, LINC00941 has been shown to modulate multiple signaling pathways in GI cancers, exerting influence on critical tumor-related biological functions both *in vivo* and *in vitro* 35, 37, 40-42, 47, such as proliferation, migration, invasion, epithelial-mesenchymal transition (EMT), tumor growth, and metastasis. Additionally, its expression has been correlated with clinical features and survival outcomes 35, 42, 45, 47, highlighting its potential as a biomarker for GI cancers.

Here, we review the molecular mechanisms, cellular processes, and clinical implications associated with LINC00941 in GI cancers. We hope this will identify knowledge gaps and outline future research directions in this field. By providing a detailed assessment, this review aims to highlight the potential of LINC00941 as a promising candidate for a novel therapeutic target in GI cancers, offering new avenues to improve patient outcomes.

## LINC00941 in gastrointestinal cancers

**Figure 1:**
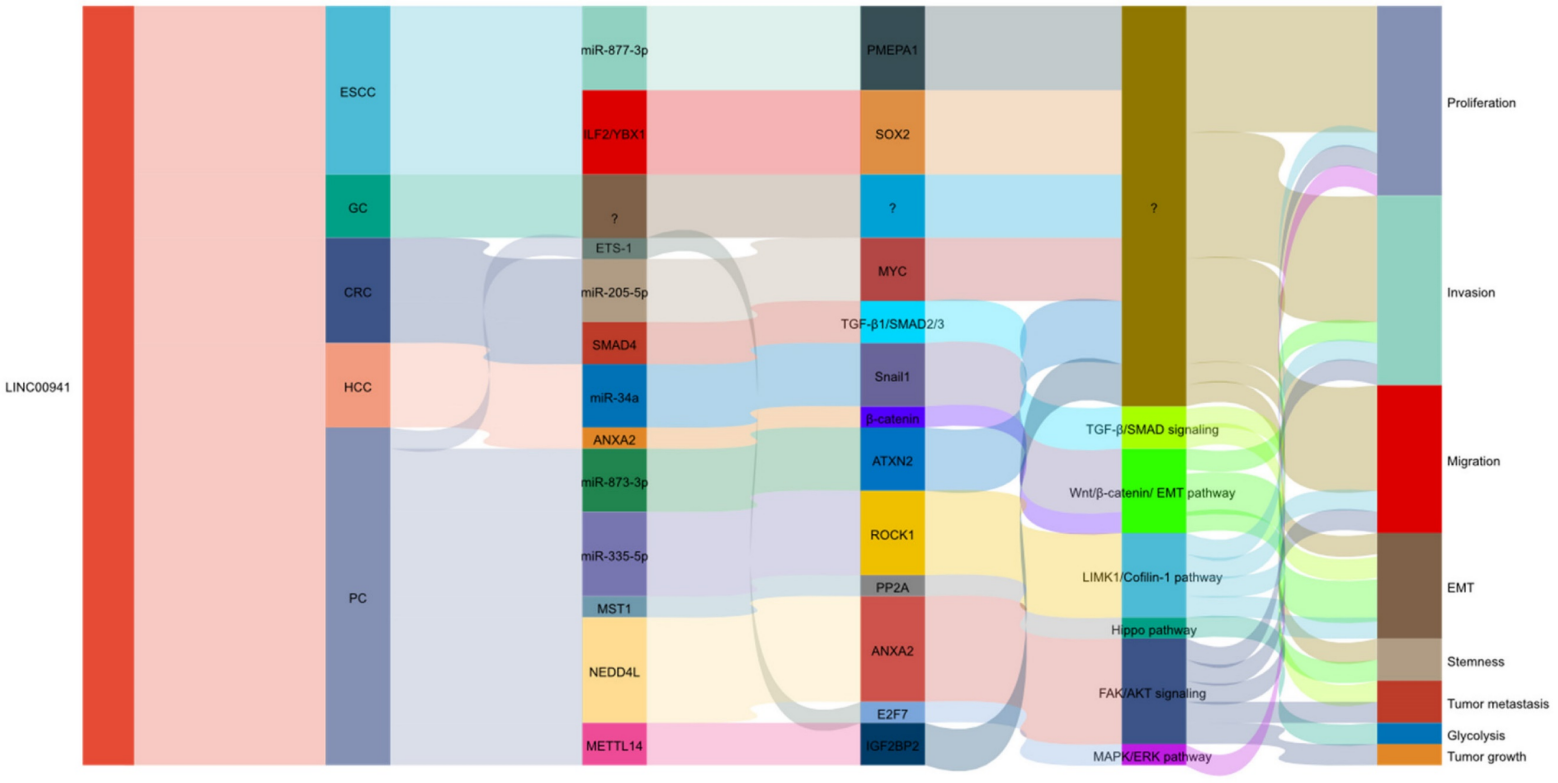
LINC00941 contributes to gastrointestinal cancer development by exerting regulatory control over various biological processes through related genes and signaling pathways.

**Figure 2:**
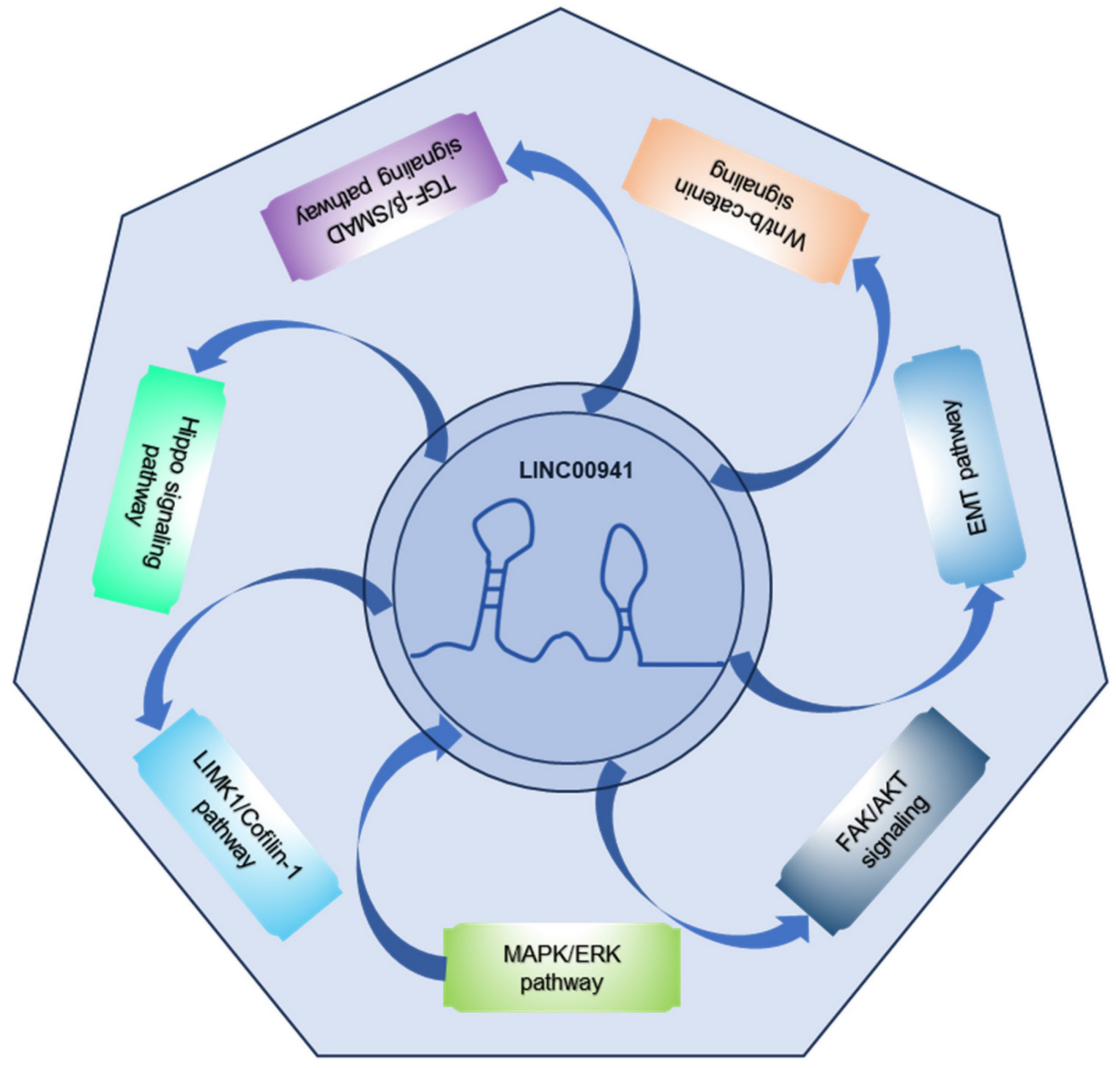
Signaling pathways involved or regulated by LINC00941.

LINC00941 has been studied in various GI cancer types, including squamous cell carcinoma (ESCC) 35, 39, gastric cancer (GC) 45, 46, colorectal cancer (CRC) 41, 44, hepatocellular carcinoma (HCC) 47, and pancreatic cancer (PC) 36, 37, 40, 42, 43, 48, 49. Research have shown that LINC00941 plays a regulatory role in GI development by influencing key molecules and genes involved in a series of biological processes, such as proliferation, invasion, migration, and EMT (**Figure 1**). In GI cancers, dysregulated expression of LINC00941 has been observed in cancer cells and tissues, and LINC00941 has been found to function as an oncogene and promote tumorigenesis and progression in GI cancers. Functionally, LINC00941 exerts regulatory functions in specific gene expression by interacting with related proteins at the transcriptional and/or post-transcriptional levels. LINC00941 also acts as a competing endogenous RNA (ceRNA) for binding to microRNA (miRNA), thereby upregulating gene expression. Furthermore, LINC00941 is implicated in the activation of multiple signaling pathways, such as TGF-β/SMAD signaling pathway, Wnt/β-catenin signaling, and Hippo signaling pathway, or regulated by signaling pathways, like MAPK/ERK pathway, as shown in **Figure 2**. The functional implications of LINC00941 have been explored through *in vitro* and/or *in vivo* experiments, shedding light on its critical involvement in GI cancer progression. In the subsequent sections, we discuss the specific outcomes and implications of these experiments in detail.

## Studies utilizing cancer cell lines

**Figure 3:**
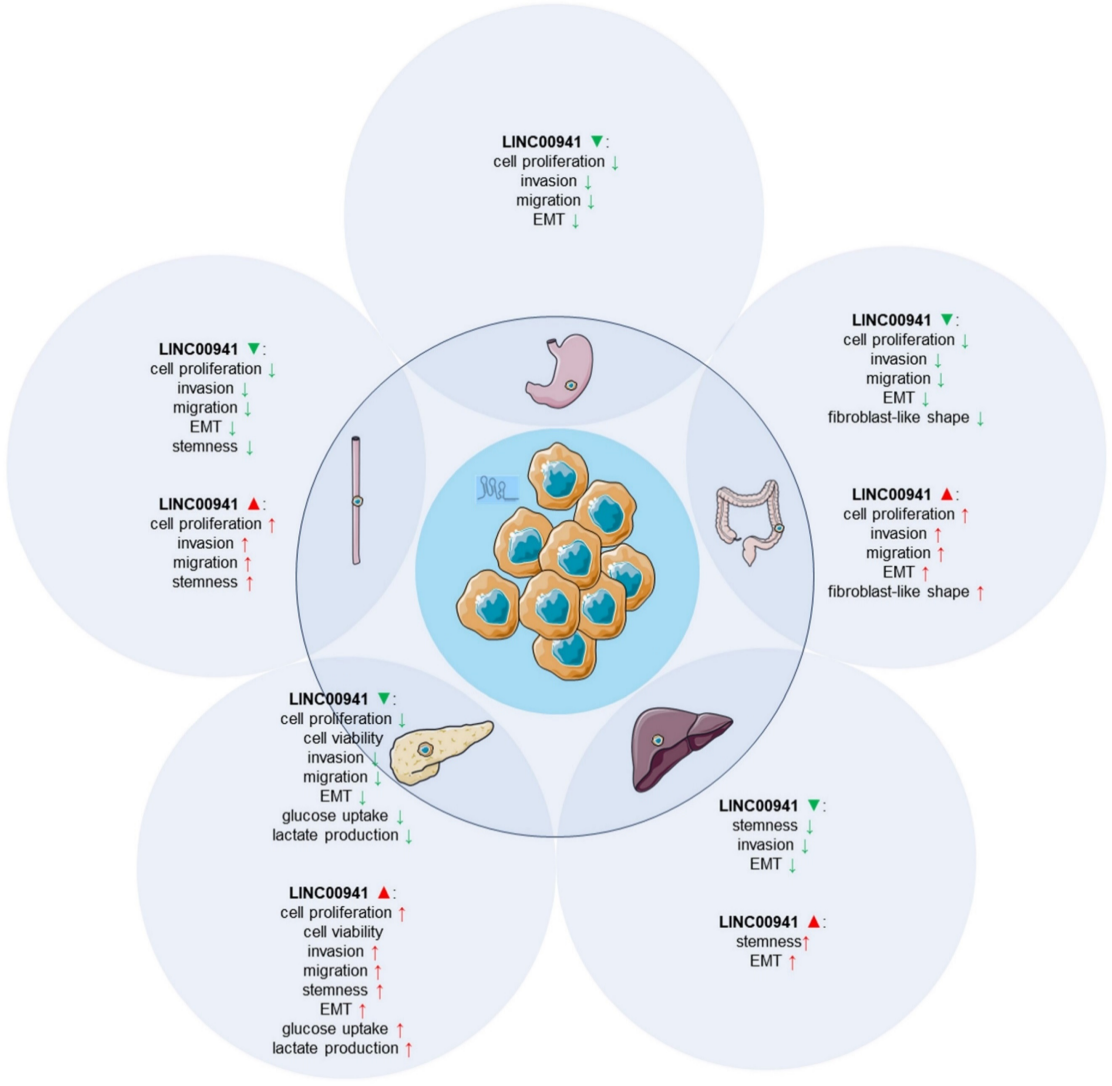
Functional impact of LINC00941 overexpression or silencing *in vitro*.

*In vitro* studies investigating the role of LINC00941 in various GI cancer cell types have provided valuable insights into its functional effects. A range of cell-based functional assays, including proliferation, migration, invasion, tumor sphere formation, and glycolysis, were conducted to elucidate the impact of LINC00941. **Table 1** presents the expression patterns of LINC00941 in GI cancer cell lines, along with their respective subcellular localizations and the corresponding functional assays employed. **Figure 3** illustrates the functional roles of LINC00941 when either silenced or/and overexpressed in GI cell lines.

**Figure 4:**
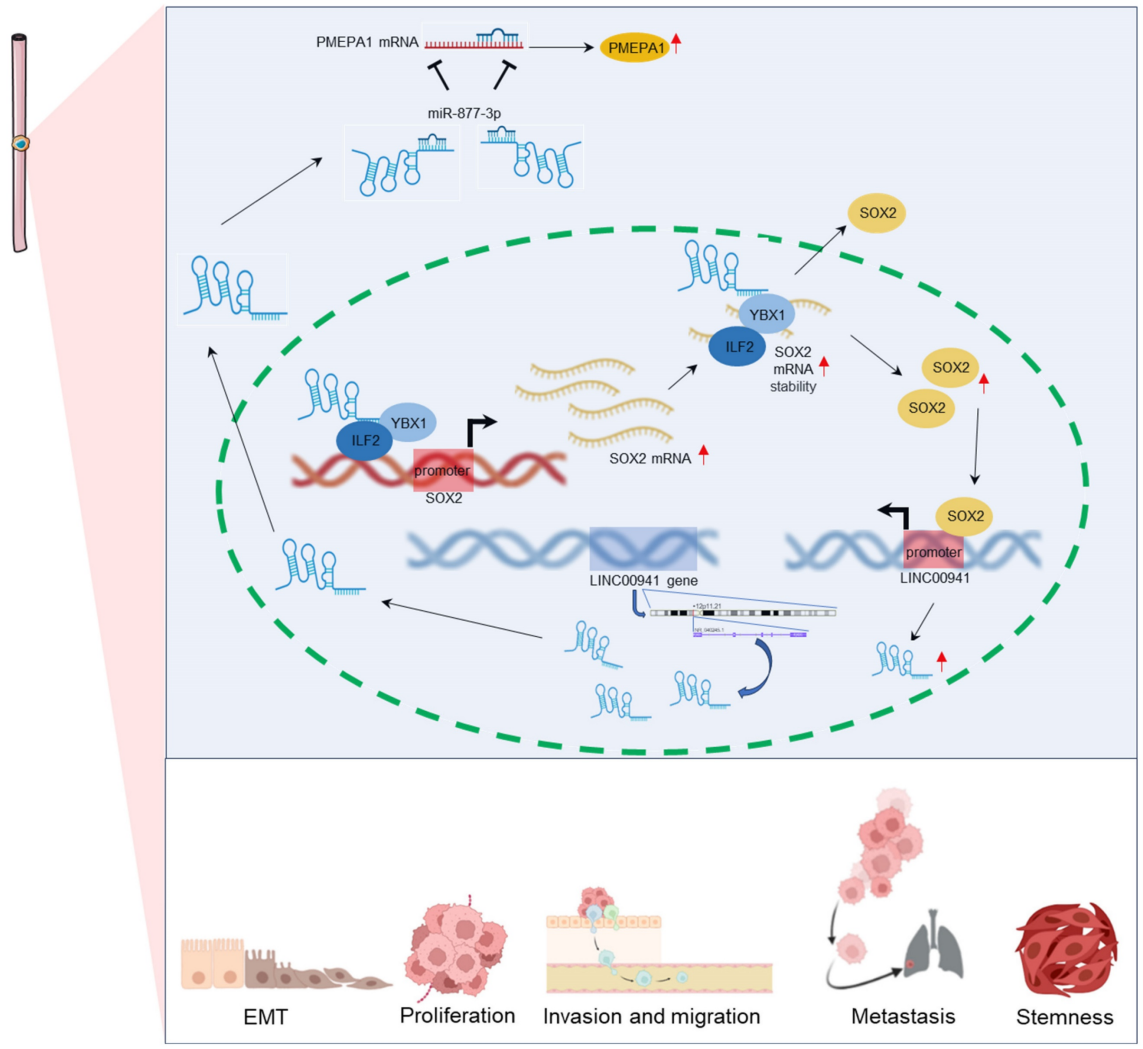
Regulatory mechanisms of LINC00941 in esophageal squamous cell carcinoma.

In ESCC cell lines, studies by Zhang et al. 39 and Lu et al. 35 reported that LINC00941 is significantly highly expressed in cancer cells compared to human esophageal epithelial cells. And LINC00941 was found to promote ESCC cell proliferation, invasion, migration, and stemness while inhibiting EMT. Mechanistically (**Figure 4**), the oncogenic role of LINC00941 involves interactions with the miR-877-3p/PMEPA1 axis 39 or a positive feedback loop involving LINC00941-ILF2/YBX1-SOX2 35.

**Figure 5:**
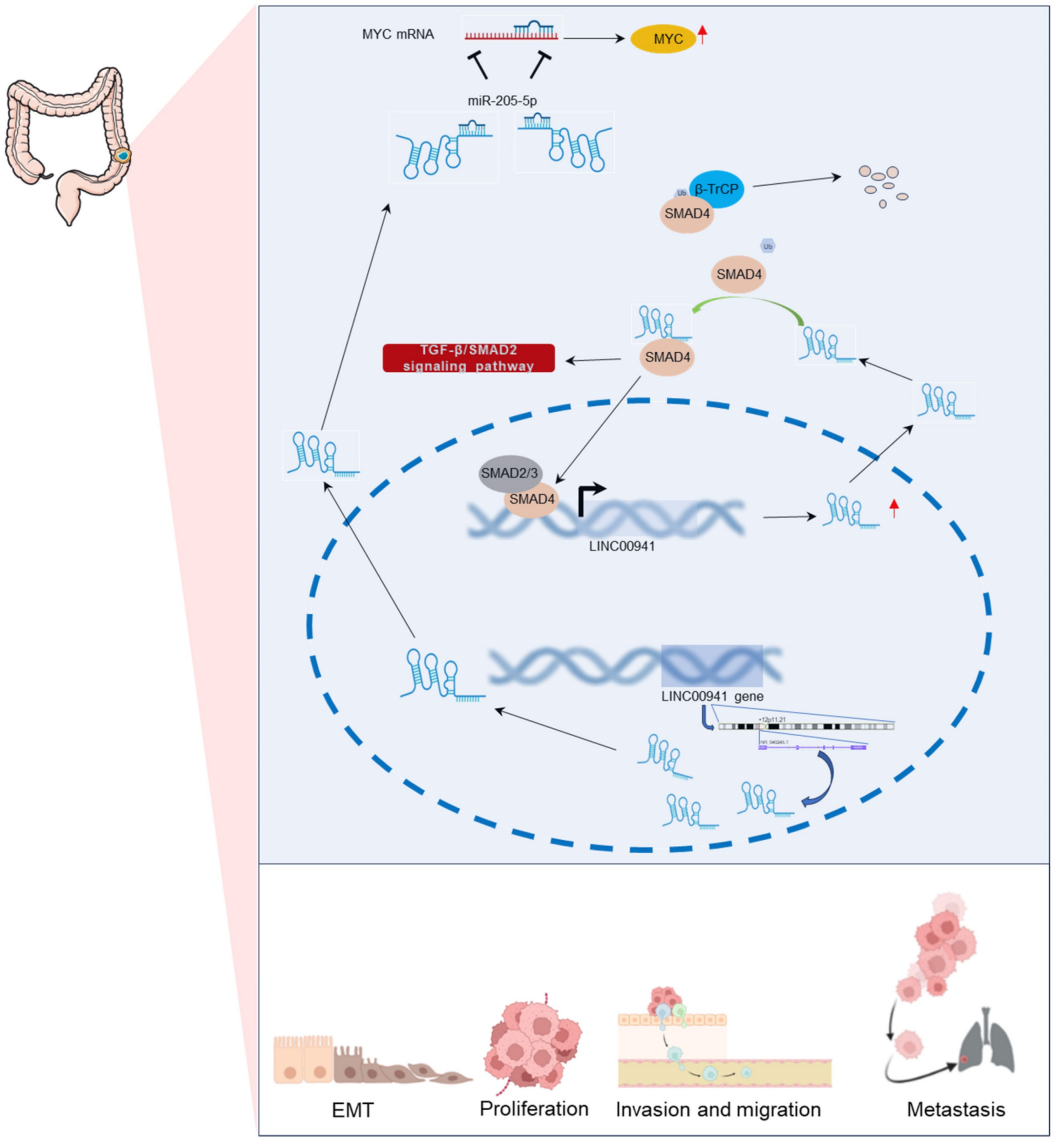
Regulatory mechanisms of LINC00941 in colorectal cancer.

In GC cell lines, LINC00941 has the potential to modulate the metastatic properties of GC cells, particularly migration and invasion, by influencing the EMT process 45. LINC00941 is up-regulated in CRC cells and contributes to cell proliferation, migration, and invasion by sponging miR-205-5p and upregulating MYC expression 44. Notably, Wu et al 41 found that LINC00941 activates EMT in CRC by binding to the SMAD4 protein, preventing degradation, and activating the TGF-β/SMAD signaling pathway. The regulatory mechanism of LINC00941 in CRC is displayed in **Figure 5.**

For HCC 47, LINC00941 is highly expressed in tumor spheres and highly invasive HCC cells. Overexpression of LINC00941 accelerates the activation of EMT and enhances the malignant capacity of HCC cells, while its suppression produces opposite effects. Mechanistically, LINC00941 promotes EMT by bounding ANXA2 and activating Wnt/β-catenin signaling or by acting as a competing endogenous RNA for miR-34a, leading to Snail1 upregulation.

**Figure 6:**
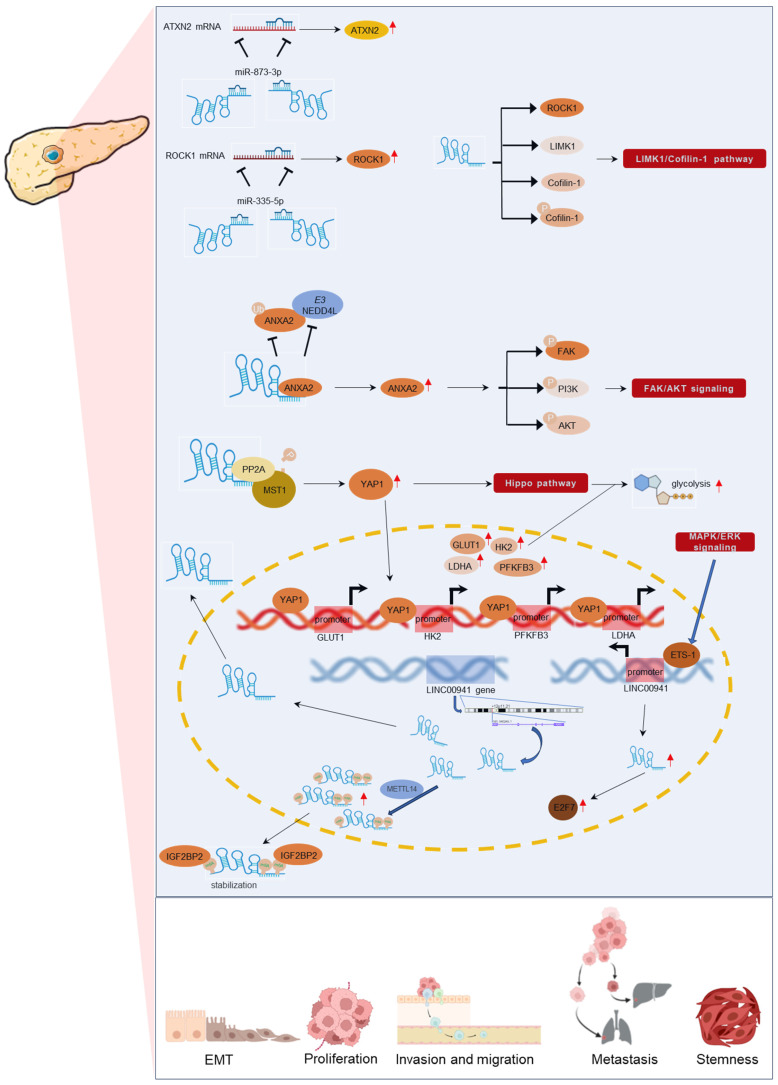
Regulatory mechanisms of LINC00941 in pancreatic cancer.

Regarding to PC, LINC00941 is highly expressed in cancer cell lines and mainly located in the cytoplasm 37, 40, 42. Overexpression of LINC00941 promotes cell proliferation, viability, invasion, migration, EMT, and stemness, while downregulation leads to the opposite effects. Specifically, LINC00941 could stimulate metastasis and EMT by competitively binding miR873-3p to upregulate ATXN2 43 or act as a molecular sponge for miR-335-5p, thereby promoting ROCK1 upregulation, and activating LIMK1/Cofilin-1 pathway 42. Wang et al 37 observed that LINC00941 could increase PC cell proliferation and enhance metastatic properties by upregulated ANXA2 and activating FAK/AKT signaling. And according to the study by Lu et al 36, LINC00941 was upregulated by METTL14 in an m6A-dependent way, contributing to the migration and invasion of PC cells. LINC00941 was also identified as the most upregulated lncRNA among MAPK-associated in PC 48, showing a positive relationship with cell proliferation in MIA PaCa-2 and AsPC-1 48. Furthermore, Xu et al 40 reported that LINC00941 promotes PDAC cancer cell growth by enhancing aerobic glycolysis, and mechanistically, LINC00941 interacts with MST1, leading to Hippo pathway activation, and enhancing glycolysis in PDAC 40. A detailed regulatory mechanism of LINC00941 in pancreatic cancer is presented in **Figure 6**.

## Studies using murine animal models

**Figure 7:**
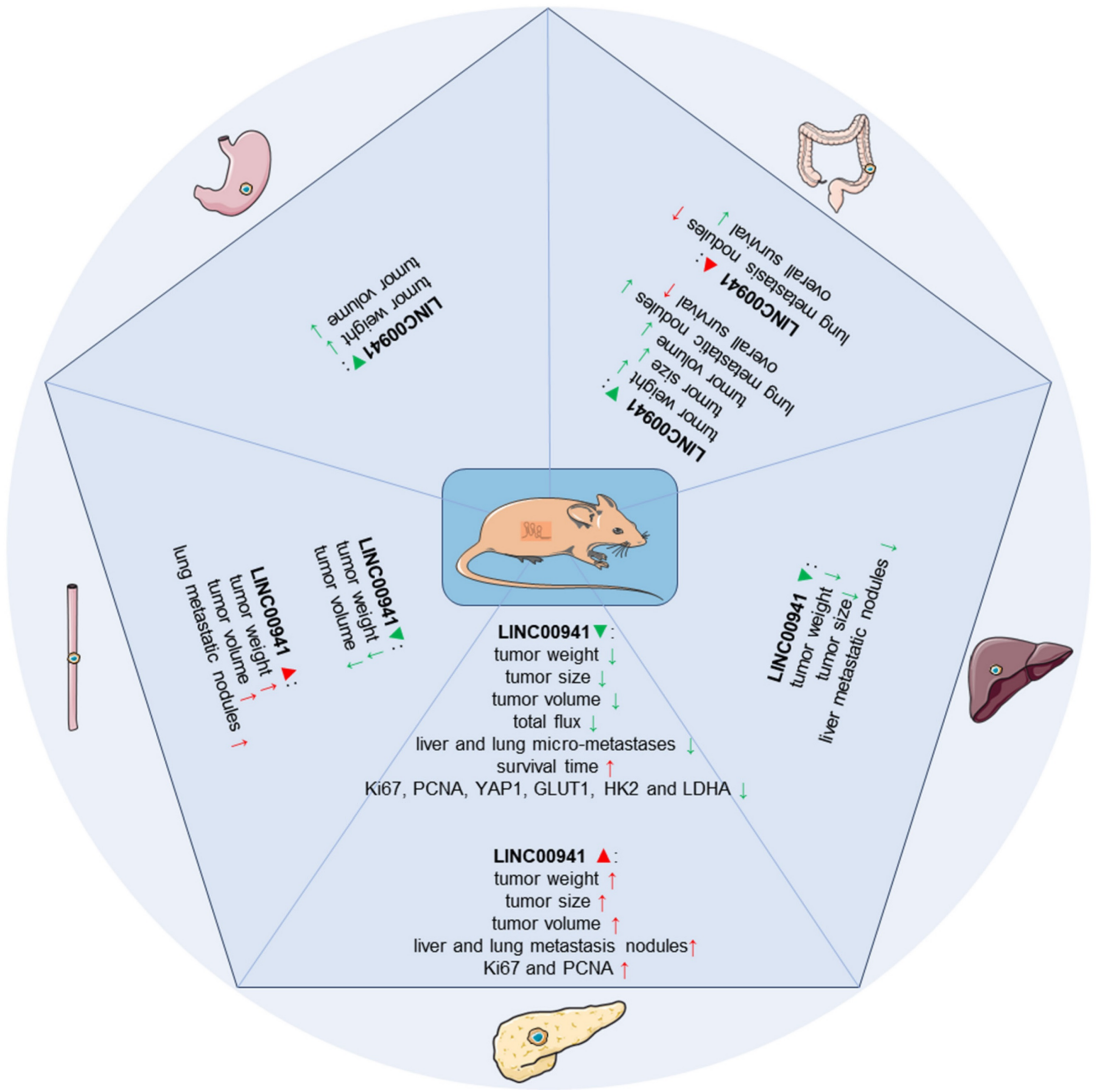
Functional impact of LINC00941 overexpression or silencing *in vivo*.

A number of research teams have extensively investigated the functional implications of manipulating LINC00941 expression in tumor development using xenograft models, including subcutaneous tumor models, tumor metastasis models, and orthotopic transplantation models, as detailed in **Table 2** and **Figure 7**. Consistent with *in vitro* studies, LINC00941 has been reported to have oncogenic roles in gastrointestinal cancers.

In the case of ESCC 35, 39, *in vivo* knockdown of LINC00941 significantly attenuated tumor progression, while its overexpression promoted tumor growth and metastasis. In GC 45, modulation of LINC00941 affected tumor growth, resulting in smaller tumors and reduced weight upon knockdown. Similarly, in CRC 41, 44, LINC00941 knockdown impaired tumor growth, metastasis, and the formation of lung nodules, while its overexpression increased the number of lung metastatic nodules. Depletion of LINC00941 in HCC suppressed xenograft formation and metastasis, leading to smaller tumors and a decreased number of liver nodules 47. In PC 36, 37, 40, 42, 43, 48, 49, LINC00941 played a significant role in promoting tumor growth, metastasis, and the expression of proliferation and glycolysis-related markers. Conversely, depletion of LINC00941 resulted in reduced tumor size, growth rate, metastases, and improved prognosis. These collective findings highlight the pivotal role of LINC00941 in tumor development and underscore its potential as a promising therapeutic target in various gastrointestinal cancers.

## Studies with clinical patient samples

The expression of LINC00941 showed significant up-regulation in various GI cancers. This up-regulation was confirmed through multiple methodologies, such as database analysis, reverse transcription quantitative polymerase chain reaction (RT-qPCR), and *in situ* hybridization (ISH). A summary of LINC00941 expression and its associations with pathological features, prognosis, and diagnostic value in clinical settings of GI tumors is presented in** Table 3**.

In ESCC 35, 39, elevated expression of LINC00941 in tumor tissues suggests its potential role in ESCC progression. Furthermore, high expression levels of LINC00941 were significantly associated with poor five-year survival rates in ESCC patients 35. Similarly, in GC 45, 46, LINC00941 was up-regulated in cancer tissues and exhibited a significant correlation with clinicopathological features, including invasion depth, lymphatic metastasis, and TNM stage. These findings highlight the diagnostic and prognostic potential of LINC00941 in GC, as it can discriminate between different pathological tissue samples and predict patient survival time. In CRC, LINC00941 displayed amplified expression in cancer tissues and showed significant associations with clinical characteristics such as tumor invasive depth, metastasis, and stage (41). Notably, patients with higher expression levels of LINC00941 experienced significantly shorter overall survival times 41, indicating its potential as an independent risk factor for poor prognosis in CRC. Consistent with the other cancers, HCC specimens exhibited consistently high expression levels of LINC00941 compared to adjacent non-tumor tissues. Kaplan-Meier survival analysis further demonstrated a correlation between elevated LINC00941 expression and poor survival outcomes in HCC patients 47, 50. Moreover, elevated levels of LINC00941 were detected in the serum of HCC patients (35), suggesting its potential as a non-invasive diagnostic biomarker for HCC. In PC, LINC00941 expression was found to be higher in pancreatic cancer tissues, and its localization was primarily observed in the cytosol of tumor cells. LINC00941 expression positively correlated with advanced pathological features 36, 40, 42, and higher expression levels of LINC00941 were associated with unfavorable prognoses in PC patients 36, 37, 40, 42, 43, 48, 49, further highlighting its significance as a prognostic biomarker for PC. Overall, the consistent upregulation of LINC00941 in various gastrointestinal cancers and its association with clinical features and patient outcomes shows its potential as a valuable biomarker for diagnosis, prognosis and risk assessment of these diseases.

## Future perspectives and conclusion

LINC00941 has recently attracted considerable attention in gastrointestinal oncology research. Based on available literature, LINC00941 has been studied in five common types of human gastrointestinal cancers, namely, esophageal, gastric, colorecta, hepatocellular, and pancreatic cancers. *In vitro* experiments have revealed its oncogenic role in many cellular processes (**Figure 3**), including promoting proliferation, migration, invasion, EMT, and enhancing stemness, and facilitating glycolysis. Moreover, murine animal models also have been employed. In xenograft models, LINC00941 silencing has consistently shown inhibitory effects on tumor progression, reducing tumor growth and metastasis. Conversely, overexpression of LINC00941 has been associated with enhanced tumor growth and metastatic potential (**Figure 7**). At the molecular level, LINC00941 mainly functions as a ceRNA, upregulating downstream gene expressions by competing for specific microRNAs (miR-877-3p, miR-205-5p, miR-34a, miR-873-3p, and miR-335-5p) 39, 42-44, 47. Additionally, it also regulates gene expression at the transcriptional and/or post-transcriptional levels through interactions with related proteins, and further contributing to the initiation and progression of GI cancers via activation of critical signaling pathways (Hippo signaling pathway, TGF-β/SMAD signaling pathway, Wnt/b-catenin signaling, EMT pathway, FAK/AKT signaling, and LIMK1/Cofilin-1 pathway) 37, 40-42, 47. Collectively, these findings provide valuable insights into the functional role of LINC00941 in tumor development, highlighting its potential as a target for therapeutic interventions.

In addition, numerous studies have consistently demonstrated that the expression of LINC00941 is upregulated in comparison to normal tissues and cell lines, implying its potential involvement in gastrointestinal tumorigenesis. Furthermore, LINC00941 expression is closely linked to advanced tumor characteristics in various types of gastrointestinal cancer, suggesting its potential role in promoting tumor progression and metastasis. The heightened expression of LINC00941 has been associated with an unfavorable prognosis in ESCC, CRC, HCC and PC. This underscores LINC00941's potential as an independent risk factor for poor prognosis in these cancer types. In terms of diagnosis, LINC00941 has shown potential as a diagnostic biomarker for GC and HCC. Overall, the upregulation of LINC00941 in gastrointestinal cancers and its correlation with clinicopathological features, as well as its value as both a prognostic and diagnostic biomarker, highlight its significance in comprehending the pathogenesis and clinical management of these cancers. Nevertheless, further research is required to validate the diagnostic and prognostic value of LINC00941 in gastrointestinal tumors within a larger and more diverse patient population.

Despite the progress made, the functional role and clinical value of LINC00941 in gastrointestinal tumors remain incompletely understood, posing several challenges and unanswered questions that warrant exploration. Firstly, the potential involvement of LINC00941 in other gastrointestinal tumors, such as gallbladder cancer, gastrointestinal stromal tumors, small intestine tumors, and bile duct cancer, remains unexplored so far and requires investigation. Additionally, the relationship between LINC00941 and chemotherapy and radiotherapy resistance in gastrointestinal tumors is currently unclear. Although existing studies have shown that a variety of long non-coding RNAs are associated with tumor therapy resistance 51-55, whether LINC00941 is involved in the resistance in the treatment of gastrointestinal tumors is still unknown, but it is worth revealing. Moreover, there is a need for further investigation into the expression of LINC00941 in fluid tissues, such as serum, and its potential as a prognostic and diagnostic marker in gastrointestinal tumors. Non-coding RNAs in fluid tissues have shown promise as tumor markers and diagnostic indicators 56-58. It is of great significance to explore the expression level of LINC00941 in liquid tissues and its correlation with the prognosis and diagnosis of patients with gastrointestinal tumors.

**Figure 8:**
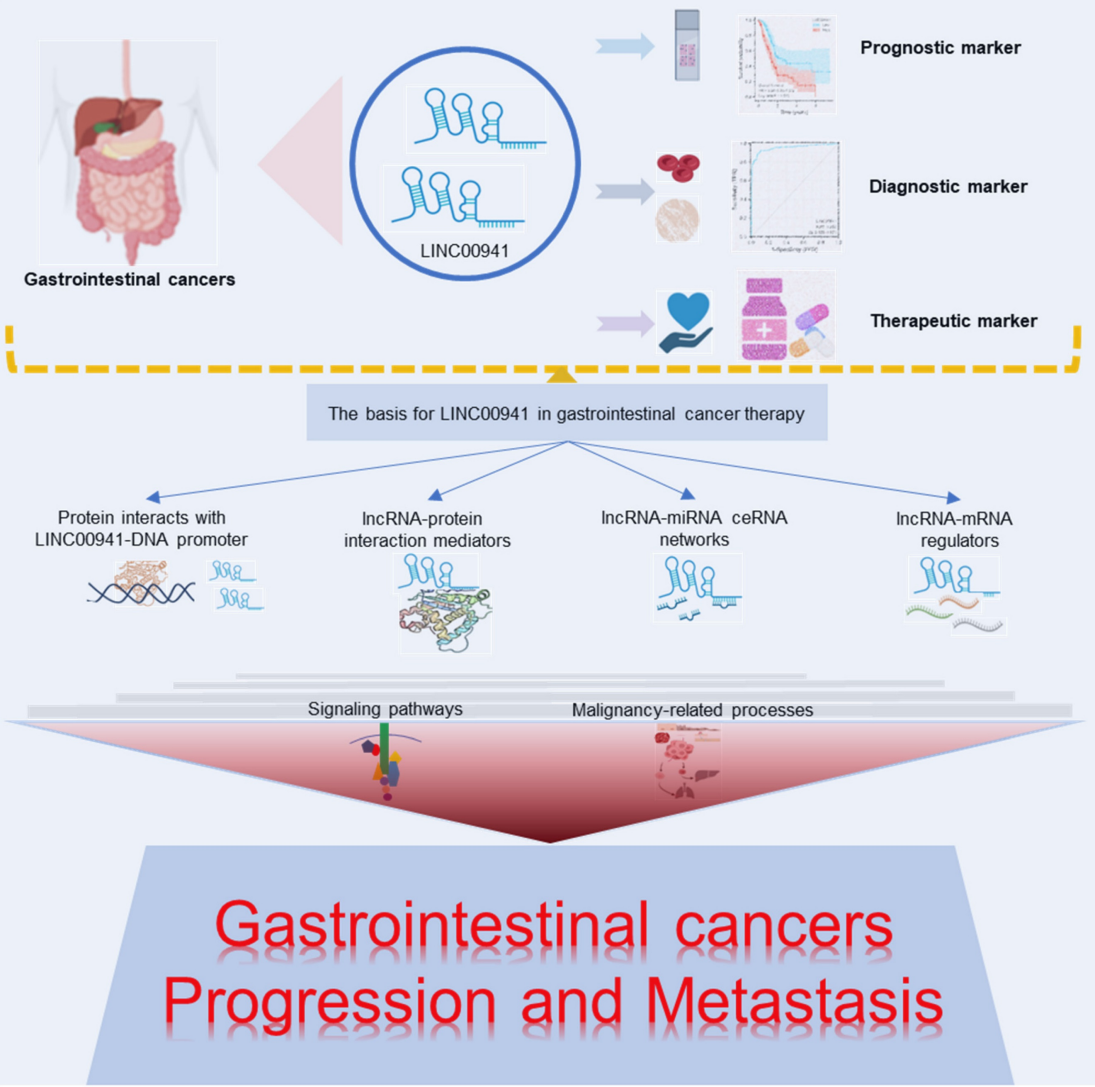
LINC00941 is involved in the progression of gastrointestinal cancer and its application prospect as a biomarker and therapeutic target of gastrointestinal cancer.

In summary, LINC00941 may hold promise as a tumor marker and therapeutic target in GI cancers (**Figure 8**). It has emerged as a valuable tool for clinical prognosis and diagnosis, offering potential avenues for targeted interventions in GI tumors. The expression of LINC00941 can be regulated by related proteins interacting with its DNA promoter, and LINC00941 can participate in the lncRNA-miRNA ceRNA network and regulate lncRNA-protein or lncRNA-mRNA interactions. These mechanisms collectively contribute to the significant involvement of LINC00941 in the development and progression of GI tumors, influencing multiple oncogenic signaling pathways and malignancy-related behaviors. These findings underscore the potential of LINC00941 as a predictive biomarker and an attractive target for GI cancers. Further experimental investigations are necessary to fully elucidate the underlying mechanisms of LINC00941 and establish robust theoretical support. Future clinical studies focusing on LINC00941 are imperative to uncover innovative ideas and approaches for GI cancer diagnosis, risk stratification, and treatment.

## Author contributions

Every author has significantly contributed to the conception, design, execution, and analysis of this study, participated in drafting or revising the manuscript, approved the final version to be published, agreed on the journal for submission, and collectively assumes responsibility for the work's integrity and accuracy.

## Figures and Tables

**Table 1:** Summary of experiments in cancer cell lines.

Cancer type	Cells			*In vitro* experiment cell lines	Function assays	Ref.
	Expression	Comparison	Subcellular localization			
ESCC	Up	(KYSE-510, KYSE-30 and Eca-109 cells) vs. human esophageal epithelial cells (HEEC)	/	KYSE-510, KYSE30	colony formation assay, CCK-8 assay, EdU assay, transwell invasion and migration assay	39
	Up	(KYSE-170, KYSE-150, TE-1, and YES-2) vs. HEEC	mainly in nucleus	KYSE-170, TE-1	MTS assay, colony formation assay, wound healing assay, transwell chambers assay, tumor sphere formation	35
GC	/	/	/	MKN45, AGS	CCK-8 assay, colony formation assay, cell migration and invasion assay	45
CRC	Up	(LS174T, HCT116, CT26, HCT8, HCT29, SW480 and LoVo) vs. colonic epithelial cells (NCM460)	/	HCT116, LoVo	CCK8 assay, transwell assay	44
	Up	(HT-29, HCT-116, SW480, SW620, and LoVo cells) vs. immortalized normal colon epithelial cells (FHC cells)	/	HCT116, LoVo	transwell assay, invasion and migration assays, morphological changes	41
HCC	Highly expressed in invasive HCC cells	HCC cell lines: LM3>MHCC97-L> SMMC-7721> HepG2>Hep3> Huh7	mainly in cytoplasm	LM3,97L,Huh7, PLC, HepG2, PHCC, Hep3B	transwell coculture system, tumor sphere formation	47
PC	Up	(AsPC-1, BxPC-3, MIA PaCa-2, PANC1, and Capan-2) vs. pancreatic ductal epithelial cell line (HPDE)	mainly in cytoplasm	MIA PaCa-2, PANC1	CCK8 assay, colony-formation assay, wound-healing assay, transwell migration and invasion assay	37
	/	/	/	MIA PaCa-2, AsPC-1, PCI-35	MTT proliferation assay	48
	/	/	In cytoplasm and nucleus	PANC-1, AsPC-1	wound healing assay, transwell migration and invasion assay	36
	Up	(PANC-1, PK-9, BxPC-3, SW1990) vs. pancreatic ductal epithelial cell line (HPDE6-C7)	/	BxPC-3, PANC-1	CCK-8 assay, EdU assay, transwell migration and invasion assay	43
	Up	(AsPC-1, BxPC-3, PANC-1, MIA PaCa-2, and Capan-2) vs. HPDE	mainly in cytoplasm	MIA PaCa-2, PANC-1	transwell migration and invasion assay, CCK-8 assay, colony formation assay	42
	Up	(AsPC-1, BxPC-3, CFPAC-1, HPAC, PANC1, and SW1990) vs. HPDE	mainly in cytoplasm	PANC-1, SW1990, BxPC-3, CFPAC-1	CCK-8 assay, colony formation assay, 3D 'on-top' assay, seahorse assay, glucose oxidase assay	40
	Up	(BxPC-3, AsPC-1, Pan-02) vs. hTERT-HPNE	/	/	/	49

ESCC: Esophageal Squamous Cell Carcinoma, GC: Gastric Cancer, CRC: Colorectal Cancer, HCC: Hepatocellular Carcinoma, PC: Pancreatic Cancer, "/ ": Indicates missing or not applicable data.

**Table 2:** Summary of experiments in murine models.

Cancer type	Animal models	Groups	Objectives	Ref.
ESCC	BALB/c nude mice (4-6 weeks old)	sh-NC and sh-LIN00941 groups	Tumor weight, tumor volume	39
	Subcutaneous xenograft model (6-week-old male BALB/c-nude mice)	LINC00941-overexpressing and control groups	Tumor weight, tumor volume	35
	Metastatic model (6-week-old male BALB/c-nude mice)	LINC00941-overexpressing and control groups	Lung metastatic nodules	
GC	Subcutaneous xenograft model (BALB/c nude mice)	sh-NC and sh-LIN00941 groups	Tumor weight, tumor volume	45
CRC	Nude mice xenograft model (BALB/c mice, male, 6-8 weeks old)	sh-NC and sh-LIN00941 groups	Tumor weight, tumor size, tumor volume, lung metastatic nodules	44
	Metastatic model (BALB/C nude mice, male, 6 weeks old)	sh-NC and sh-LIN00941 groups; LINC00941-overexpressing and control groups	Lung metastatic nodules, overall survival	41
HCC	Subcutaneous xenograft (nude mouse)	sh-NC and sh-LIN00941 groups	Tumor weight, liver metastatic nodules	47
PC	Subcutaneous xenograft (six-week-old BALB/c nude mice)	Vector, Lv-LINC00941, Lv-LINC00941 + sh-ANXA2, Lv-LINC00941 + PF-562271 30mg/kg/d	Tumor weight, tumor volume, IHC: Ki67 and PCNA	37
	Metastasis model	Vector, Lv-LINC00941, Lv-LINC00941 + sh-ANXA2, Lv-LINC00941 + PF-562271 30mg/kg/d	Liver and lung metastasis nodules	
	Metastasis model (BALB/c nude mouse)	sh-NC and sh-LIN00941 groups	Liver metastatic nodules	36
	Subcutaneous xenografts model	sh-NC and sh-LIN00941 groups	Tumor weight, tumor size, tumor volume, IHC and qpcr: Ki67 and PCNA	42
	Metastatic tumor model	sh-NC and sh-LIN00941 groups	Liver and lung micro-metastases, survival time	
	Subcutaneous xenograft model (BALB/c athymic nude mice)	sh-NC and sh-LIN00941 groups	Tumor weight, tumor volume, IHC staining: PCNA, YAP1, GLUT1, HK2, and LDHA	40
	Orthotopic implantation model (C57BL/6J mice)	Control, sh-LIN00941, Gemcitabine, sh-LIN00941+ Gemcitabine	Overall survival, total flux, IHC staining: PCNA, YAP1, GLUT1, HK2, and LDHA	

ESCC: Esophageal Squamous Cell Carcinoma, GC: Gastric Cancer, CRC: Colorectal Cancer, HCC: Hepatocellular Carcinoma, PC: Pancreatic Cancer.

**Table 3:** Summary of LINC00941 expression and its associations with pathological features, prognosis, and diagnostic value in clinical samples of gastrointestinal cancers.

Cancer type	Expression (sample)	Clinical feature	Disease endpoint	Survival analysis	Diagnostic analysis	Biomarker	Ref.
ESCC	Up (tissue)	/	/	/	/	/	39
	Up (tissue)	/	OS	Kaplan-Meier curve	/	Prognosis	35
GC	Up (tissue)	tumor depth, distant metastasis	OS	Kaplan-Meier curve	ROC curve (normal vs. tumors) (M1 vs. M0 samples)	Prognosis/ Diagnosis	45
	Up (tissue)	invasion depth, lymphatic metastasis, TNM stage	/	/	ROC curve (normal vs. tumors)	Diagnosis	46
CRC	Up (tissue)	tumor invasive depth, lymph node metastasis, distant metastasis	/	/	/	/	44
	Up (tissue)	lymph node metastasis, AJCC stage	OS	Kaplan-Meier curve, Univariate and Multivariate analysis	/	Prognosis	41
HCC	Up (tissue)	/	OS	Kaplan-Meier curve	/	Prognosis	47
	Up (tissue)	/	OS	Kaplan-Meier curve	/	Prognosis	50
	Up (serum)	/	/	/	ROC curve (HCC patients vs. healthy controls)	Diagnosis	38
PC	Up (tissue)	/	OS	Kaplan-Meier curve, Univariate and Multivariate analysis	/	Prognosis	37
	Up (tissue)	/	OS	Kaplan-Meier curve	/	Prognosis	48
	Up (tissue)	AJCC stage, N stage	OS	Kaplan-Meier curve	/	Prognosis	36
	Up (tissue)	/	OS, DFS	Kaplan-Meier curve	/	Prognosis	43
	Up (tissue)	tumor diameter, lymphatic metastasis	OS, DFS	Kaplan-Meier curve	/	Prognosis	42
	Up (tissue)	tumor size, tumor stage	OS	Kaplan-Meier curve	/	Prognosis	40
	/ (tissue)	/	OS	Kaplan-Meier curve	/	Prognosis	49

ESCC: Esophageal Squamous Cell Carcinoma, GC: Gastric Cancer, CRC: Colorectal Cancer, HCC: Hepatocellular Carcinoma, PC: Pancreatic Cancer, OS: Overall Survival; DFS: Disease-Free Survival, "/ ": Indicates missing or not applicable data

## Abbreviations

GI: Gastrointestinal
ncRNAs: non-coding RNAs
lncRNAs: long non-coding RNAs
lncRNA-MUF: MSC-upregulated factor
LINC00941: Long Intergenic Non-Protein Coding RNA 941
EMT: epithelial-mesenchymal transition
ESCC: esophageal squamous cell carcinoma
GC: gastric cancer
CRC: colorectal cancer
HCC: hepatocellular carcinoma
PC: pancreatic cancer
ceRNA: competing endogenous RNA
miRNA: microRNA
RT-qPCR: reverse transcription quantitative polymerase chain reaction
ISH: *in situ* hybridization
